# Lymph node involvement is associated with overall survival for elderly patients with non-metastatic gallbladder adenocarcinoma

**DOI:** 10.3389/fsurg.2024.1414870

**Published:** 2024-07-23

**Authors:** Jianhao Huang, Yanyu Qiu, Xuesong Bai, Xiaodong He

**Affiliations:** Department of General Surgery, State Key Laboratory of Complex Severe and Rare Diseases, Peking Union Medical College Hospital, Peking Union Medical College & Chinese Academy of Medical Sciences, Beijing, China

**Keywords:** gallbladder cancer, overall survival, elderly patients, nomogram, SEER

## Abstract

**Background:**

Lymph node involvement is recognized as a prognostic factor for patients with gallbladder cancer. However, the N stage varied from different editions of the American Joint Committee on Cancer (AJCC) TNM Classification. Our objective was to investigate the impact of lymph node involvement on overall survival in elderly patients with non-metastatic gallbladder adenocarcinoma.

**Methods:**

Patients older than 65 years with non-metastatic gallbladder adenocarcinoma were identified from the SEER data. We used Cox proportional hazard regression analysis to select the independent risk factor. A nomogram was built to identify the 1-, 3-, and 5-years’ prognostic impact. Univariate and multivariate models were used to examine the correlation of overall survival (OS) with the number of metastatic nodes.

**Results:**

A total of 1,654 patients (706 with and 948 without nodal involvement) were included. Cox proportional hazard regression analyses found that age, gender, tumor size, lymph node involvement, and surgical options were risk factors for the prognosis and were integrated into the nomogram. After adjustment, OS was compromised for patients who receive surgery with nodal involvement [hazard ratio (HR), 2.238; *P* < 0.01]. Furthermore, after adjustment the presence of more than two metastatic lymph nodes was associated with decreased OS (HR, 3.305; *P* < 0.01).

**Conclusions:**

Our results suggest that lymph node involvement is associated with compromised survival in elderly patients. A nomogram was developed to predict the prognosis of gallbladder cancer. A change point of more than two metastatic lymph nodes seems to carry prognostic significance, calling for closer monitoring of elderly patients with gallbladder cancer with involvement of increased number of lymph nodes.

## Introduction

Gallbladder cancer (GBC) is a common biliary tract malignancy, also characterized by a very poor prognosis (1). Based on global cancer statistics, fewer than 5,000 new cases are diagnosed with GBC each year, but approximately 2,000 cases die from GBC (2, 3). Because the early stage of the disease often presents asymptomatic, patients who presented with abdominal pain or jaundice usually had progressive disease with a worse prognosis (4). Recently, with improvements in surgical strategies, survival for patients with GBC has significantly improved, and 5-year overall survival has reached 18% (5).

Gallbladder cancer mainly occurs in middle-aged and elderly patients. In a recent survey, among 44,470 patients with gallbladder cancer, 31,615 (71.1%) patients were older than 65 years (6). With increasing age, rates steadily rose and the highest rate was observed among people 85 years of age (7). Consequently, further investigation should focus on the prognosis of elderly patients with GBC. For early-stage gallbladder cancers, surgery treatment is the primary treatment option, involving removal of the gallbladder. Lymph node resection or resection of involved adjacent organs mainly depends on the TNM stage of the tumor. For non-metastatic gallbladder cancers, radical surgery is suggested with long-term improved survival in 15%–63% (8).

Lymph node involvement is a common mode of metastasis in gallbladder cancer and an independent prognostic risk factor (9). Regional lymph node dissection is an important component of radical cholecystectomy for gallbladder cancer, and its significance mainly includes diagnosis and treatment. The N stage varied from different editions of Nevin staging system and the American Joint Committee on Cancer (AJCC) TNM Classification for Gallbladder Cancer. In the seventh edition, the determination of N stage was based on the location of the involved lymph node. N1 stage indicated the lymph nodes along the cystic duct, common bile duct, hepatic artery, and/or portal vein were involved. N2 stage was considered when the tumor metastases to the periaortic, pericaval, superior mesenteric artery, and/or celiac artery lymph nodes (10). However, in the eighth edition, the N stage depends on the number of metastatic lymph nodes. N1 stage indicated that fewer than three nearby lymph nodes were involved. If four or more nearby lymph nodes were spread, N2 stage was determined. Consequently, a better staging strategy may need further investigation (11).

We used the SEER database to examine the impact of lymph node metastases and the absolute number of metastatic lymph nodes on survival for patients older than age 65 years with non-metastatic gallbladder cancer. We hypothesized that the presence of lymph node metastases and increased number of metastatic lymph nodes are associated with overall survival in patients older than 65 years with non-metastatic gallbladder cancer and that it is possible to stratify patients' relative risk of death based on the nomogram to predict 1-, 3-, and 5-year overall survival (OS) rates based on significant prognostic factors. Furthermore, we carried out external validation for this prediction model using our hospital database.

## Methods

### SEER database

The SEER database represents approximately 28% of the US population and was used to identify patients diagnosed with gallbladder adenocarcinoma (ICD-O-3 codes 8140/3) between 2004 and 2019. Variables included patient age at diagnosis, gender, year of diagnosis, survival months, and vital status. Treatment data included extent of surgery and use of radiotherapy or chemotherapy. Pathologic characteristics included tumor size, number of regional nodes examined, and number of regional nodes involved. The combined summary stage of SEER was used to determine the presence of distant metastases at diagnosis.

### Study population

Adult patients older than age 65 years with non-metastatic gallbladder cancer were included. Patients with multiple cancer diagnoses and/or distant metastases based on the combined summary stage of SEER were excluded. The study cohort included patients diagnosed up to 2019 to allow a minimum of 3 years of follow-up survival data. The study cohort was categorized into two groups: patients who are still alive and patients who had died. The other data source comprised patients with gallbladder cancer who were diagnosed with gallbladder cancer preoperatively and adenocarcinoma postoperatively, and received surgical treatment at Peking Union Medical College Hospital from 2004 to 2019. Histological assessment of tumor tissues and immunohistochemical tests were performed at the pathology department to confirm. SEER data are publicly available and only include deidentified data so that institutional review board approval was not necessary for this study. The included patients from our center were approved by the Institutional Review Board of Peking Union Medical College Hospital (S-K3900). We certify that the study was performed in accordance with the 1964 Helsinki Declaration and its later amendments.

### Statistical analyses

Descriptive statistics were computed for patient characteristics by vital status for the SEER cohorts. Wilcoxon rank-sum tests were used for continuous variables and Pearson *χ*^2^ tests were used for categorical variables. Overall survival time was estimated by the Kaplan–Meier method and long-rank test. OS was defined as the period from the date of diagnosis to the date of death from various causes. Patients alive at the date of the last contact were censored. Univariate Cox proportional hazards model was used to screen out significant prognostic variables (*P*-value <0.05) for further multivariate Cox analysis. We designed all significant variables in the multivariate Cox regression (*P*-value <0.05) as prognostic factors in the establishment of nomogram. We carefully chose variables for inclusion to ensure parsimony of the final model.

To investigate the correlation between the number of metastatic lymph nodes and survival in patients with gallbladder cancer who received surgery, proportional hazards regression model with restricted cubic splines (RCSs) was used to analyze the number of metastatic lymph nodes (12). RCSs established a model to estimate the correlation between the hazard ratio (HR) and the number of positive lymph nodes observed. We then investigated the effect of the change point of the positive lymph nodes on the relative overall survival of patients with gallbladder cancer undergoing lymph node dissection. The aim of this method is to maximize the adjusted difference on the overall survival between two groups of patients classified by the change point of positive lymph nodes.

All analyses were performed with complete information. All *P*-values reported are two-sided with the significance level set to 0.05. Statistical analyses were performed by using SPSS version 25 (IBM, Armonk, NY, USA) and R version 4.2.2.

## Results

### SEER cohort

A total of 1,654 patients met the inclusion criteria. Table 1 displays the general demographic and clinicopathological features of patients chosen from the SEER database. The population was divided into two groups: patients who were still alive and patients who had died. Male patients and patients aged ≥75 years accounted for the majority of patients who had died. Among patients who did not receive surgery, the proportion of dead patients (1.3%) was greater than that of living patients (0.2%). Tumors between 2 and 5 cm in size accounted for approximately half of both living patients (45.9%) and dead patients (47.3%). Most alive patients (80.9%) underwent radiation therapy, while no difference was found between the number of dead patients and living patients who received chemotherapy. In patients who are still alive, 72.1% of the patients show no regional lymph node involvement and 52.3% of the dead patients show regional lymph node involvement. However, the number of examined lymph nodes between the two groups are similar (5.27 ± 0.13 vs. 5.81 ± 0.16, *P* = 0.174). In addition, 140 patients from the Peking Union Medical College Hospital were investigated. Their characteristics are shown in Supplementary Table S1.

**Table 1:** Clinicopathological features of SEER cohorts.

Patient status	Alive (*n* = 653)	Dead (*n* = 1,001)	*P*-value
Age	** **		<0.01
<75 years old	382 (58.5%)	444 (44.4%)	
≥75 years old	271 (41.5%)	557 (55.6%)	
Gender	** **		0.031
Male	190 (29.1%)	342 (34.2%)	
Female	463 (70.9%)	659 (65.8%)	
Year of diagnosis	** **		<0.01
2004–2012	161 (24.7%)	568 (56.7%)	
2013–2019	492 (75.3%)	433 (43.3%)	
Surgery	** **		0.012
Yes	652 (99.8%)	988 (98.7%)	
No	1 (0.2%)	13 (1.3%)	
Tumor size	** **		0.004
<2 cm	244 (37.4%)	308 (30.8%)	
2–5 cm	300 (45.9%)	473 (47.3%)	
>5 cm	109 (16.7%)	220 (22.0%)	
Lymph node examination number	5.27 ± 0.13	5.81 ± 0.16	0.174
Lymph node involvement	** **		<0.01
Yes	182 (27.9%)	524 (52.3%)	
No	471 (72.1%)	477 (47.7%)	
Radiation therapy	** **		0.001
Yes	99 (15.2%)	220 (22.0%)	
No	554 (84.8%)	781 (78.0%)	
Chemotherapy	** **		0.272
Yes	249 (38.1%)	354 (35.4%)	
No	404 (61.9%)	647 (64.6%)	

**Figure 1:**
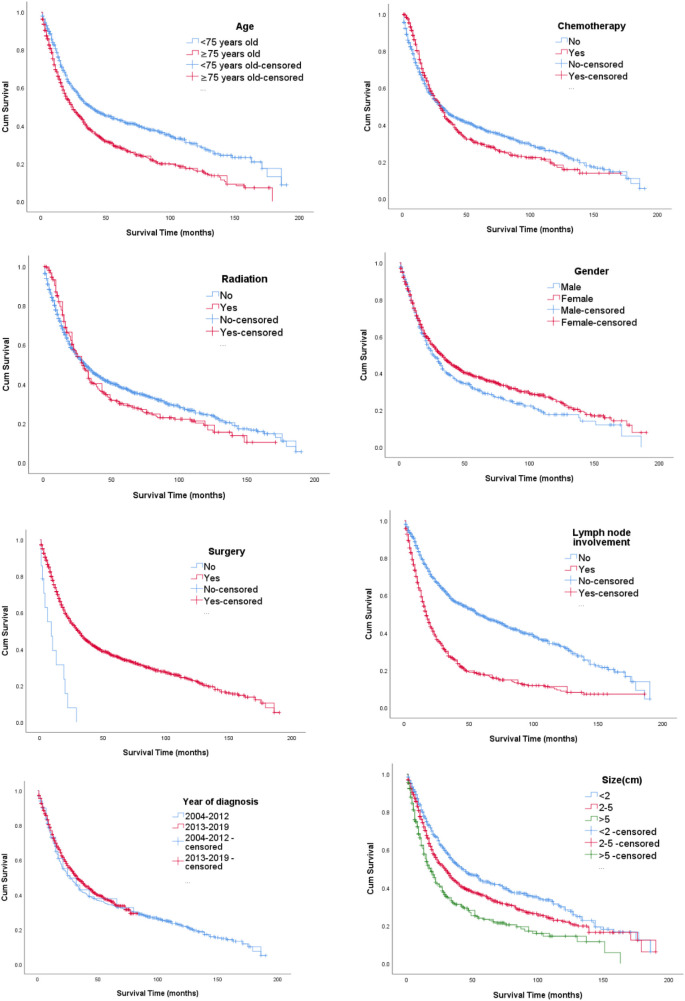
Kaplan–Meier curves of OS according to (1) age, (2) chemotherapy, (3) radiation, (4) gender, (5) surgery, (6) lymph node involvement, (7) year of diagnosis, and (8) size.

Figure 1 Kaplan–Meier curves based on age, race, gender, year of diagnosis, tumor size, lymph node involvement, radiation, chemotherapy, and surgical options. The median OS of all included patients was 34.7 months. Patients younger than 75 years (38.7 months) and who received surgery (34.9 months) had longer median survival times than patients older than 75 years (30.7 months) who received no surgery (10.1 months). Furthermore, the median survival time of patients with a tumor size less than 2 cm was 41.4 months. Also, patients with gallbladder cancer with regional lymph node involvement had worse survival with a median OS of 23.8 months. All differences were statistically significant (*P* < 0.001) through the log-rank test. However, we did not detect increased survival time in radiotherapy and chemotherapy.

**Figure 2:**
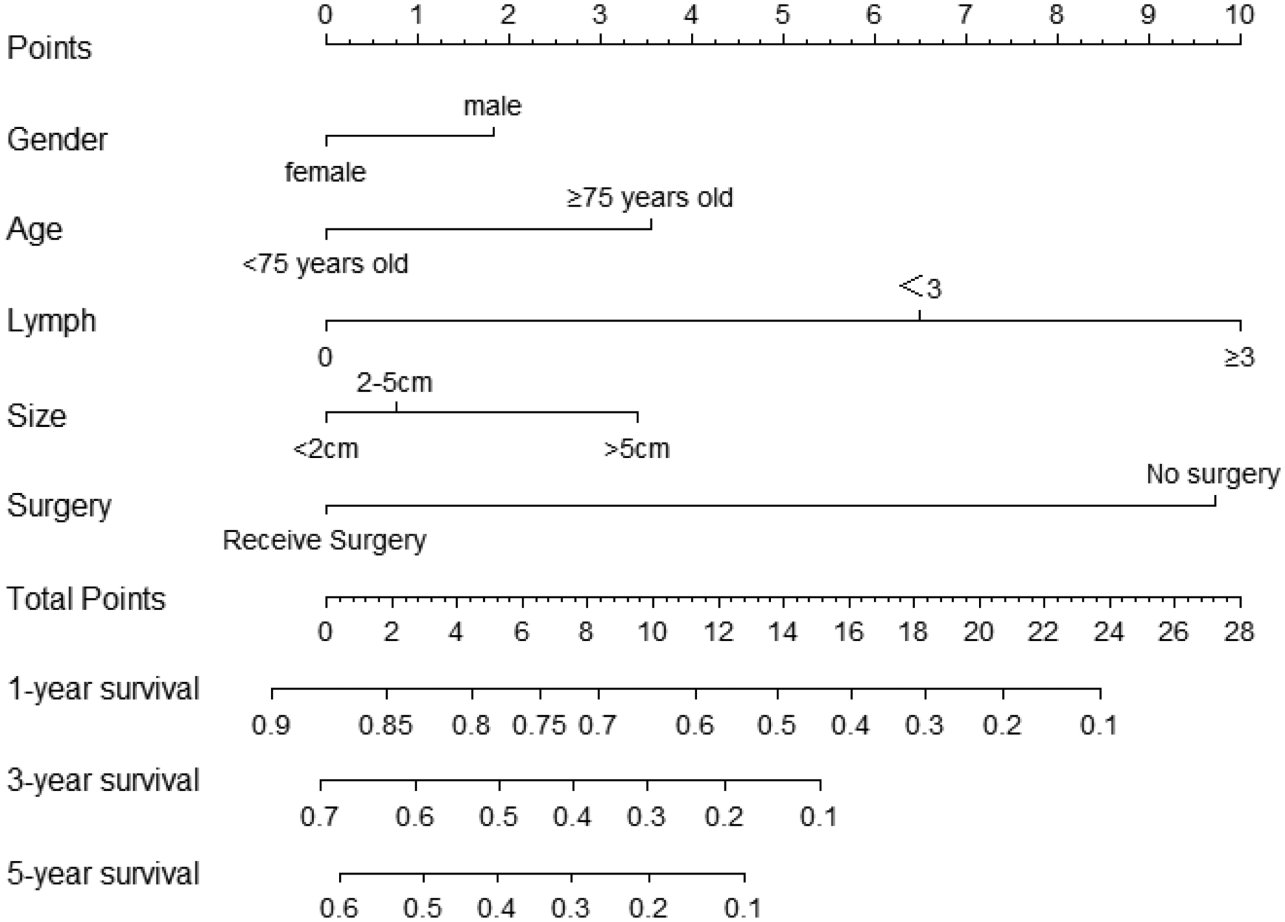
Nomograms predicting 1-year, 3-year and 5-year rates of OS. Summarizing the scores of each variables together and the total points projected on the bottom scales indicate the probabilities of 1-, 3- and 5-year overall survival.

**Figure 3:**
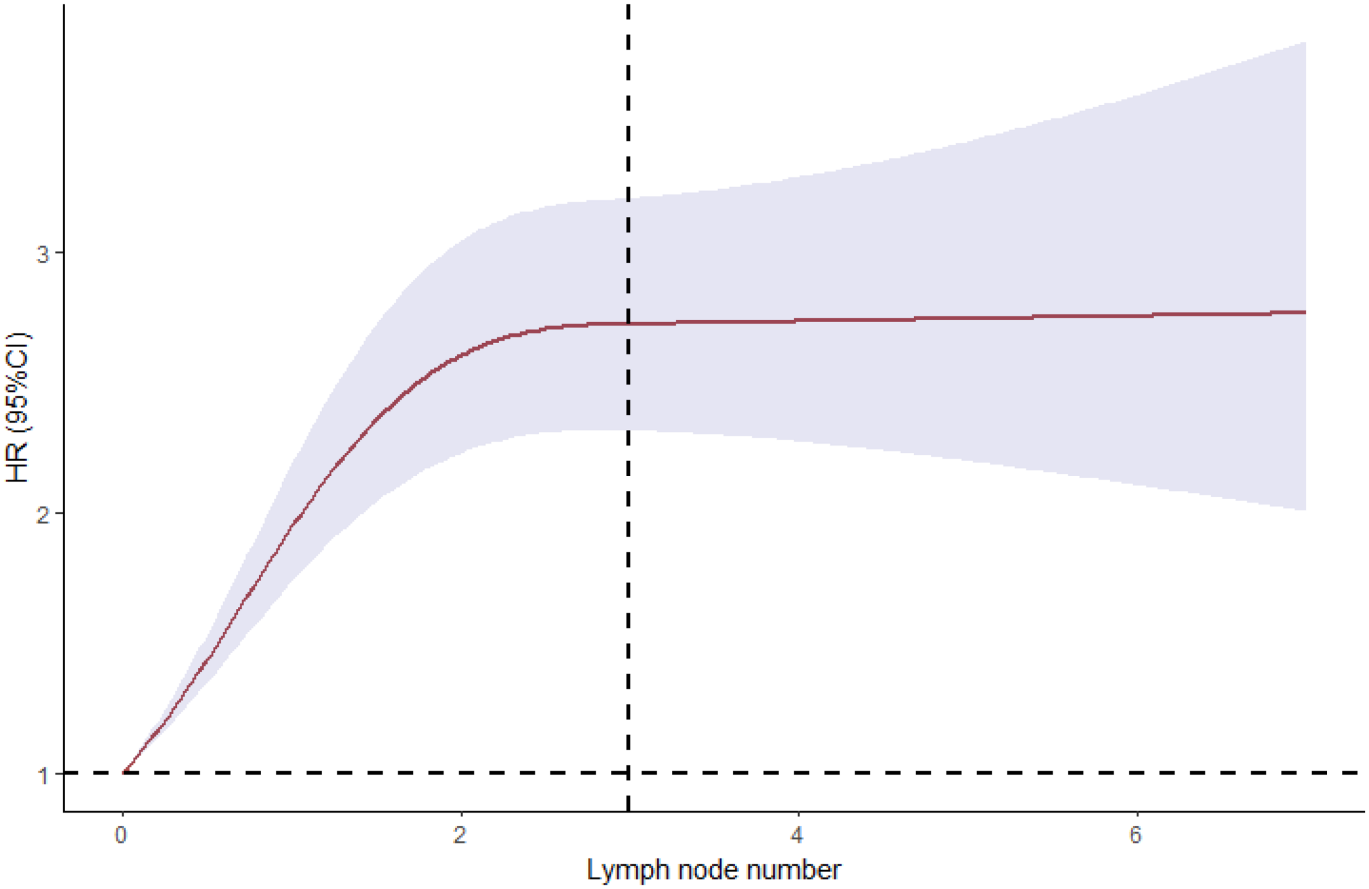
Smoothed restricted cubic spline plot of the log hazard ratio (HR) versus the number of metastatic lymph nodes. The grey zone represents the 95% CIs about the predicted HRs.

Table 2 presents the results of univariate and multivariate Cox analyses of patients with non-metastatic gallbladder cancer from the SEER database. We regarded being older than 75, male gender, no surgery treatment, regional lymph node involvement, and a tumor size greater than 5 cm to be significant risk factors for decreased survival time. Further, we included all the independent risk factors to build a nomogram for predicting 1-, 3-, and 5-year OS in elderly patients with GBC (Figure 2). As the nomogram shows, surgery and lymph nodes were the most statistically significant risk factors affecting overall survival, followed by gender, age, and tumor size. The C-index of the nomogram was 0.656 (0.638–0.674) and 0.715 (0.670–0.767) in the training and external validation cohorts, respectively. In both the training cohort and the external validation cohort, the calibration curves show that the predicted values of the nomogram agree (Figure 3).

**Table 2:** Univariate and multivariate COX regression analysis for the overall survival.

	Univariate analysis		Multivariate analysis	
	HR (95% CI)	*P*-value	HR (95% CI)	*P*-value
Age				
<75 years old	1		1	
≥75 years old	1.485 (1.311–1.683)	**<0**.**01**	1.518 (1.339–1.721)	**<0**.**01**
Gender				
Male	1		1	
Female	0.857 (0.751–0.976)	**0**.**02**	0.806 (0.707–0.920)	**0**.**001**
Year of diagnosis				
2004–2012	1			
2013–2019	0.905 (0.794–1.032)	0.136		
Surgery				
Yes	1		1	
No	3.751 (2.166–6.496)	**<0**.**01**	3.045 (1.754–5.286)	**<0**.**01**
Tumor size				
<2 cm	1		1	
2–5 cm	1.270 (1.100–1.466)	**0**.**001**	1.095 (0.947–1.267)	0.22
>5 cm	1.845 (1.550–2.195)	**<0**.**01**	1.520 (1.273–1.814)	**<0**.**01**
Lymph node involvement				
No	1		1	
Yes	2.313 (2.039–2.624)	**<0**.**01**	2.238 (1.967–2.548)	**<0**.**01**
Radiation therapy				
No	1			
Yes	1.069 (0.921–1.242)	0.38		
Chemotherapy				
No	1			
Yes	1.037 (0.910–1.182)	0.582		

The bold value indicate statistically significant.

### Lymph node metastases and survival

**Figure 4:**
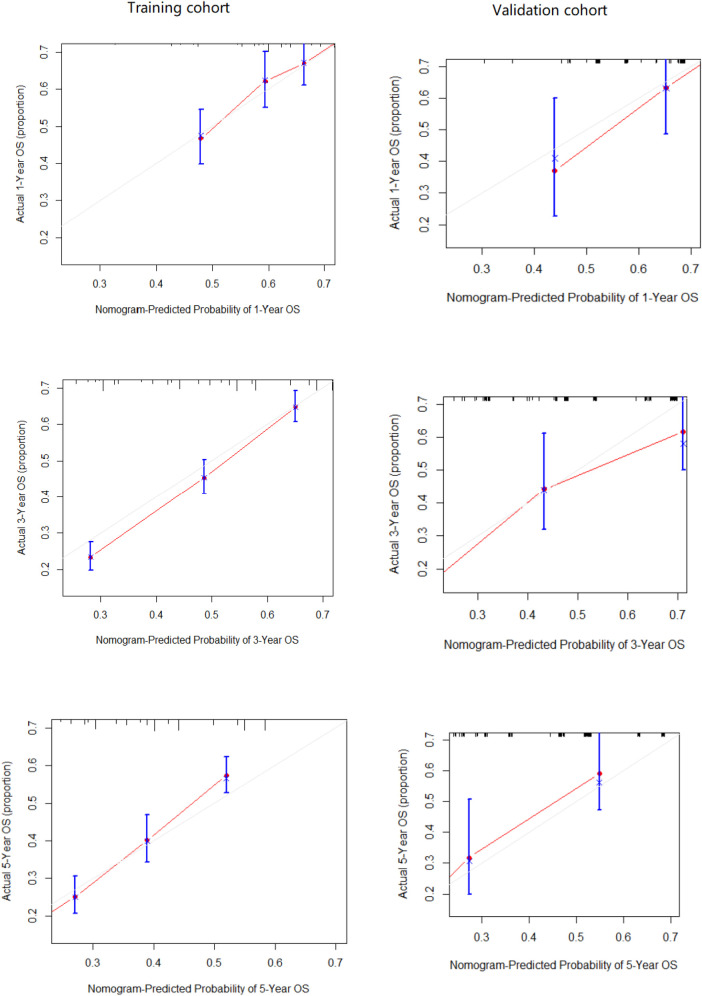
External calibration plot: 1-year, 3-year and 5-year OS nomogram calibration curves in training cohort and validation cohort.

A complete case analysis was conducted for 1,640 patients with 989 deaths. After adjustment for patient demographic and clinical and tumor characteristics, patients with regional lymph node metastases had compromised survival compared with those who did not have such metastases (HR, 2.245; 95% CI, 1.974–2.553; *P* < 0.01). We estimated the relationship between the relative risk of death and the number of metastatic lymph nodes by using a univariable model with a three-knot spline of the main variable. We observed significant effects for the overall association and the non-linear association of the number of metastatic nodes. This was also seen in the plot of HR vs. the number of metastatic lymph nodes by using the three-knot RCS. The estimated change point was 2.5 positive lymph nodes (Figure 4). By using linear splines, we estimated the HR before the change point of three positive lymph nodes to be 2.182 (95% CI, 1.913–2.489; *P* < 0.01) and the HR after the change point to be 3.305 (95% CI, 2.384–4.581; *P* < 0.01) (Table 3). These results suggest that patients with lymph node metastases had a significantly increased risk of death if more than two positive lymph nodes were involved.

**Table 3:** Summary of hazards ratio results for the SEER cohorts of elderly patients with non-metastatic gallbladder cancer.

Effect	Non-adjusted HR (95% CI)	*P*-value	Adjusted HR (95% CI)*	*P*-value
Lymph node involvement vs. no lymph involvement	2.313 (2.039–2.624)	<0.01	2.245 (1.974–2.553)	<0.01
No. of involved lymph nodes				
<3	2.259 (1.986–2.569)	<0.01	2.182 (1.913–2.489)	<0.01
≥3	3.229 (2.337–4.461)	<0.01	3.305 (2.384–4.581)	<0.01

* Indicated adjusted for the effects of lymph node involvement at patient age, patient gender, surgery, tumor size.

## Discussion

Previous studies have reported that the overall survival of patients with gallbladder cancer varies with age, and elderly patients with gallbladder cancer have shorter overall survival (13–15). The incidence of gallbladder cancer also peaks in elderly patients (2, 3). Consequently, we demonstrated that the clinicopathological characteristics and the number of metastatic lymph nodes were associated with overall survival for elderly patients with non-metastatic gallbladder cancer. A nomogram was established to predict the overall survival of elderly patients with non-metastatic gallbladder cancer and validated by the single-center patients with gallbladder cancer. An increasing number of metastatic lymph nodes were associated with decreased survival. Having more than two lymph nodes involved may confer an additional risk of overall survival for elderly patients with non-metastatic gallbladder cancer. Three metastatic lymph nodes may be a better threshold to classify the lymph node stage for elderly patients with non-metastatic gallbladder cancer.

For elderly patients with gallbladder cancer, Wen et al. (16) found that age, tumor histological grade, TNM stage, surgical method, chemotherapy, and tumor size were correlated with the poor prognosis, which was quite similar to our results. T stage and the tumor size account for the greatest effect on the prognosis of the 4,241 elderly patients with gallbladder cancer. In another study concerning non-metastatic gallbladder cancer, 904 patients were enrolled and age, gender, histology, T stage, and number of examined lymph nodes were considered as the prognostic factors. In our study, surgery and the number of lymph nodes involved had important role in the prognosis of gallbladder cancer. Due to limited surgery information provided by the SEER database, we subsequently focused on the role of the number of lymph nodes involved. Although the prognostic significance of lymph node metastases is recognized for patients with gallbladder cancer, the classification of the N stage is still controversial (17, 18). Both the number of metastatic lymph nodes and the location of the metastatic lymph nodes have been considered as thresholds for N-stage classification (10, 11). Also, the latest version of the N stage was based on the number of positive LNs rather than the location.

We demonstrated that lymph node metastases had significantly increased risk of death if more than two positive lymph nodes were involved. Previous studies have shown that the ratio of metastatic lymph nodes is an independent predictor for the prognosis of patients with lymph node positive gallbladder cancer (19–21). The impact of the number of metastatic lymph nodes on survival has not been well-defined (22). Several indexes, including the number of metastatic lymph nodes, the log odds of metastatic lymph nodes, and the lymph node ratio, are introduced to investigate relationship between the lymph node involvement and the prognosis of the gallbladder cancer. The log odds of metastatic lymph nodes and lymph node ratio are advocated by Amini et al. (21), with C-indexes of 0.621 and 0.615, respectively. The lymph node ratio, defined as the number of metastatic lymph nodes-to-the number of retrieved lymph nodes, equal to 0.15, stratified the prognosis of patients with lymph node–involved gallbladder cancer, while the number of metastatic lymph nodes did not affect the prognosis (19). However, Chen et al. (22) developed several models to predict prognosis and found that prediction based on the number of metastatic lymph nodes had the best accuracy of 88.15% in the tree-augmented naïve Bayesian model, a C-index of 0.763 in the Cox proportional hazards regression model, and an area under curve of 0.872 in the binary logistic regression model. The study also found that the involvement of four or more lymph nodes significantly impacts the prognosis. For our study, the involvement of more than two lymph nodes significantly impacted the prognosis in the specific gallbladder cancer groups, with adjustment for patient, tumor, and treatment features. With the improvement of surgical technology, lymph node dissection has become safer, and therefore, lymph node dissection with at least six nodes retrieved is advocated (17). Attention should be given to patients with higher number of lymph nodes involved.

Our study has several limitations that should be considered. The SEER uses coding methods to record the clinicopathological characteristics and treatment strategy. Details regarding the extent of lymph node involvement and surgery were not available. Consequently, conclusions regarding the exact location of the lymph nodes and surgical strategies cannot be drawn. In addition, the SEER database mainly includes American patients and validation data are only from a single center, thus, a multicenter study is still necessary. Other factors including serum biomarkers and genetic information are missed in the SEER database, so the predictive nomogram may need further refinement.

## Conclusion

By using nationally representative databases, we demonstrate that the presence of lymph node metastases in patients older than age 65 years with gallbladder cancer bears a worse prognosis with compromised overall survival. A nomogram was developed based on the SEER data to predict the prognosis of those patients with gallbladder cancer, with external validation. This study also provides information regarding the impact of the number of metastatic lymph nodes on survival. These findings call for a better N-stage classification of elderly patients with gallbladder cancer with increased number of lymph nodes involved.

## Data Availability

The raw data supporting the conclusions of this article will be made available by the authors, without undue reservation.
