# Supplementary material for: Lymph node involvement is associated with overall survival for elderly patients with non-metastatic gallbladder adenocarcinoma
Source: Front Surg. 2024 Jul 23;11:1414870. doi: 10.3389/fsurg.2024.1414870 (PMC11300228; doi:10.3389/fsurg.2024.1414870)
Supplement: Supplementary file 1 [file Table1.docx]

**Supplement 1. Clinicopathological characteristics of single center gallbladder cancer patients**

|  | Alive(n=55) | Dead(n=85) |
| --- | --- | --- |
| **Age** |  |  |
| <75 years old | 31 | 41 |
| ≥75 years old | 24 | 44 |
| **Gender** |  |  |
| Male | 17 | 30 |
| Female | 38 | 55 |
| **Surgery** |  |  |
| Yes | 55 | 83 |
| No | 0 | 2 |
| **Tumor Size** |  |  |
| <2cm | 21 | 20 |
| 2-5cm | 28 | 50 |
| >5cm | 6 | 15 |
| **Lymph node involvement** |  |  |
| Yes | 15 | 42 |
| No | 40 | 43 |
